# Influences of Cutting Speed and Material Mechanical Properties on Chip Deformation and Fracture during High-Speed Cutting of Inconel 718

**DOI:** 10.3390/ma11040461

**Published:** 2018-03-21

**Authors:** Bing Wang, Zhanqiang Liu, Xin Hou, Jinfu Zhao

**Affiliations:** 1 School of Materials Science and Engineering, Shandong University, Jinan 250061, China; 2 Key Laboratory of High Efficiency and Clean Mechanical Manufacture of MOE, School of Mechanical Engineering, Shandong University, Jinan 250061, China; houxin@mail.sdu.edu.cn (X.H.); sduzhaojinfu@gmail.com (J.Z.); 3 Key National Demonstration Center for Experimental Mechanical Engineering Education, Shandong University, Jinan 250061, China

**Keywords:** chip formation, constitutive and fracture parameters, sensitivity analysis, high speed machining, Inconel 718

## Abstract

The paper aims to investigate the influences of material constitutive and fracture parameters in addition to cutting speed on chip formation during high-speed cutting of Inconel 718. Finite element analyses for chip formation are conducted with Johnson–Cook constitutive and fracture models. Meanwhile, experiments of high-speed orthogonal cutting are performed to verify the simulation results with cutting speeds ranging from 50 m/min to 7000 m/min. The research indicates that the chip morphology transforms from serrated to fragmented at the cutting speed of 7000 m/min due to embrittlement of the workpiece material under ultra-high cutting speeds. The parameter of shear localization sensitivity is put forward to describe the influences of material mechanical properties on serrated chip formation. The results demonstrate that the effects of initial yield stress and thermal softening coefficient on chip shear localization are much more remarkable than the other constitutive parameters. For the material fracture parameters, the effects of initial fracture strain and exponential factor of stress state on chip shear localization are more much prominent. This paper provides guidance for controlling chip formation through the adjustment of material mechanical properties and the selection of appropriate cutting parameters.

## 1. Introduction

Nickel alloys have excellent properties, including high strength, high fracture toughness, superior corrosion resistance, and the ability to work at high temperatures [1]. These admirable properties have made nickel alloys widely used alloys in petrochemical equipment, power stations, pollution control apparatus, nuclear and marine applications, the aerospace and automotive areas, etc. [2]. The majority of machinery parts made from nickel alloys need to be manufactured with varied machining processes, such as turning, milling, and drilling.

Despite the increased demand for nickel alloys in the engineering sector, the application of them is usually limited by their poor machinability. Much research has focused on investigating the machining mechanism of nickel alloys covering different aspects, such as the chip formation mechanism, machined surface integrity, cutting force and temperature, and tool wear and tool failure. [3,4]. Because of the specific physical and mechanical properties of nickel alloys, particularly their high strength and inferior thermal properties, serrated chips are generated for a wide range of cutting speeds. Considering that chip formation has significant effects on variation of the cutting temperature, cutting force, and tool wear, it is vitally important to reveal the influence regularities of essential factors that control the chip formation process. Moreover, previous research has also proven that chip morphology affects the micro-topography of a machined surface in high-speed cutting [5]. Chip serration is prone to cause micro-waves on the machined surface and then increase the machined surface’s roughness.

The chip formation process is determined by the mechanical properties of the workpiece material and the machining conditions, of which the material is the essential factor and the conditions are the induction factor. Maruda et al. [6] investigated the influence of cooling condition on tool wear and chip formation during the turning of AISI 1045 steel, and they found that machining with a minimum quantity of cooling lubrication is beneficial for reducing tool wear and facilitating chip removal from the cutting zone. Kuczmaszewski et al. [7] assessed the safety and effectiveness for the milling of AZ91HP magnesium alloy from the viewpoint of chip fragmentation, and the conclusion was drawn that increases in feed rate and cutting speed can promote fragmented chip formation. Many researchers have focused on the serrated chip formation mechanism, and three main theories have been proposed: the adiabatic shear theory, the periodic crack theory, and the mixed theory of adiabatic shear combined with ductile fracture [8]. However, the influence of the dynamic mechanical behavior of the workpiece material on chip formation under the coupled effects of thermal and mechanical factors is still poorly understood. In addition, the nonlinear characteristics of loading conditions and localized plastic deformation within cutting areas lead to highly complex theoretical analyses which are difficult to carry out sometimes. It is more practicable to investigate material deformation behavior during the chip formation process with the aid of the finite element method.

Different simulation methods have been used to research serrated chip formation with either implicit or explicit software packages, and various material models have been adopted covering material constitutive models and damage or fracture models [9]. The material constitutive models include the empirical ones (such as the power function constitutive model and the Johnson–Cook (JC) constitutive model) and physical ones (such as the Zerilli–Armstrong model and the Mechanical Threshold Stress model) [10]. The commonly used material fracture models mainly include the equivalent fracture strain model, the JC fracture model, the Rice–Tracey model, and the Cockcroft–Latham fracture model. [11]. Some modified or new constitutive and fracture models have also recently been used in analysis of the chip formation process. Denguir et al. [12] developed a new constitutive model considering the effects of stress state and microstructure evolution based on the JC constitutive model. They predicted surface integrity parameters during orthogonal cutting of OFHC (oxygen-free high-conductivity copper), including the residual stresses, dislocation density, and grain size, with the new material model. A TANH (Hyperbolic TANgent) constitutive model developed on the basis of the JC model was proposed and its applicability was validated for predicting serrated chip formation during machining of Ti6Al4V [13]. A new material fracture model, known as the Teng–Wierzbicki model [11], was proposed considering the effects of rate dependency, temperature, and damage evolution, and it has also been used to simulate the metal cutting process [14]. To perform a simulation of a cutting process accurately with the finite element method, an appropriate selection of material constitutive and fracture models is the foremost condition. With regard to the material deformation status during a machining process, the JC constitutive model and the JC fracture model have been used the most widely because the integrated effects of strain, strain rate, and temperature are taken into consideration.

Although there has been much research conducted focusing on chip formation in nickel alloys, the influences of different material models, especially the influences of material constitutive and fracture parameters, on the chip formation process have not been understood completely. Ducobu et al. [15] investigated the effects of material constitutive model and chip separation criterion on the numerical results of a Ti6Al4V machining process, which was validated with experimental results. Their research showed that the variation of cutting force is mainly affected by the material constitutive model, while the chip morphology is mainly determined by the chip separation criterion. Another recent publication of Ducobu et al. [16] compared the chip formation of Ti6Al4V obtained with twenty different sets of material constitutive model parameters, and the results demonstrated the significance of the appropriate choice for material constitutive parameters in metal cutting simulation. Shi and Liu [17] and Umbrello [18] studied the effects of different sets of material constitutive parameters on simulation results of orthogonal cutting of HY-100 steel and AISI 316L, respectively. The output results of cutting force, chip morphology, distributions of cutting temperature, and residual stress were considered in their research, and the conclusion was drawn that the simulation results were very sensitive to the adopted material constitutive model parameters, especially for the residual stresses within the machined subsurface. Wang and Liu [9,19] investigated the influences of material mechanical properties on chip formation in Ti6Al4V, which has revealed them to be the predominant factor controlling serrated chip formation.

This paper aims to investigate the influences of cutting speed and material mechanical properties on chip formation in high-speed orthogonal cutting of Inconel 718 with a hybrid method of finite element analyses and experiments. The super alloy Inconel 718 is selected as the workpiece material, which is one of the most extensively used nickel alloys. The simulation results are validated with experiments considering chip geometrical characteristics, which prove the reliability of the numerical modelling. The chip deformation and fracture behavior for Inconel 718 under varied cutting speeds are researched. The parameter of shear localization sensitivity is put forward to describe the effects of JC constitutive and fracture parameters on chip localized deformation, which can be used to assess the effects of material mechanical properties on chip formation quantitatively.

## 2. Finite Element Modelling of Orthogonal Cutting

### 2.1. Modelling Procedure

**Figure 1:**
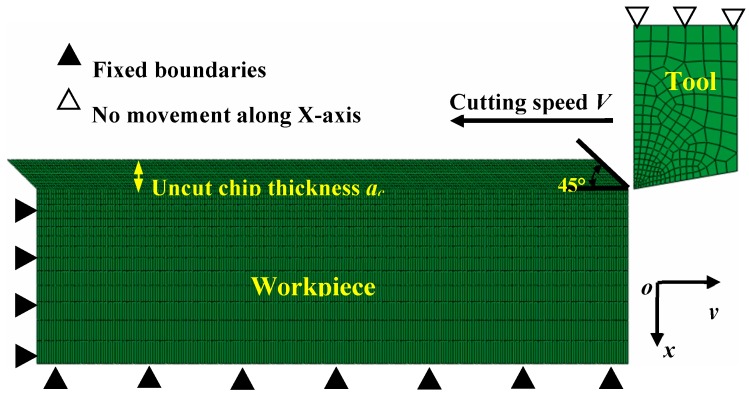
Schematic diagram of simulation model for orthogonal cutting.

The finite element software Abaqus/Explicit was used to perform the orthogonal cutting simulation of Inconel 718. Simulation models developed with Abaqus/Explicit can solve convergence issues resulting from the complexities of contact and material properties. Figure 1 displays the finite element model of orthogonal cutting created using the planar quadrilateral continuum elements (CPE4RT), which are applicable in an analysis of the coupled temperature-displacement problem. The element deletion criterion was used to ensure chip separation from the bulk workpiece material. The cutting tool was set as a rigid body and the tool rake angle was fixed as 0° in this research.

The range of cutting speed *V* was from 50 m/min to 7000 m/min, and the direction of the cutting speed was parallel with the upside of the workpiece. The uncut chip thickness *a_c_* was kept constant as 0.1 mm. The boundary conditions, including the bottom and left sides of the workpiece, were set as shown in Figure 1. The penalty contact model is adopted to define the contact property between the cutting tool and workpiece, and Coulomb’s friction law was used to describe the tool-chip interface friction as described in a previous publication [20]. A chamfer with an angle of 45° was employed at the beginning of the chip layer to eliminate excessive mesh distortion at the transient cutting-into moment.

### 2.2. Constitutive Model of Inconel 718

The JC constitutive model is widely used in finite element analyses of metal cutting because it is applicable for deformation conditions with a strain rate ranging from 10^2^ to 10^6^ s^−1^ [21]. The combined hardening and softening effects caused by the strain, the strain rate, and temperature are considered in the JC constitutive model, which is expressed by Equation (1).

(1) $$\bar{\sigma }=\underset{\mathrm{Elasto}-\mathrm{plastic}\;\mathrm{term}}{\underset{︸}{\left[A+B{\bar{\varepsilon }}^{n}\right]}}\underset{\mathrm{Viscosity}\;\mathrm{term}}{\underset{︸}{\left[1+C\ln\left(\frac{\dot{\bar{\varepsilon }}}{{\dot{\bar{\varepsilon }}}_{0}}\right)\right]}}\underset{\mathrm{Thermal}\;\mathrm{softening}\;\mathrm{term}}{\underset{︸}{\left[1-{\left(\frac{T-T_{r}}{T_{m}-T_{r}}\right)}^{m}\right]}}$$

where $\bar{\sigma }$ is the equivalent flow stress, $\bar{\varepsilon }$ is the equivalent plastic strain, $\dot{\bar{\varepsilon }}$ is the equivalent strain rate, and ${\dot{\bar{\varepsilon }}}_{0}$ is the reference strain rate. The parameters *T*, *T_m_*, *T_r_* are the current temperature, the melting temperature of the workpiece material, and the room temperature, respectively. The five JC constitutive parameters *A*, *B*, *n*, *C*, and *m* for the workpiece material of Inconel 718 are presented in Table 1. The physical and mechanical properties of Inconel 718 are specified in Table 2.

### 2.3. Chip Separation Criterion

A material fracture model was applied to realize the chip formation process. The fracture model in finite element analyses is described with the form of the cumulative damage law denoted by Equation (2), on which the JC fracture model [24] has been developed.

(2) $$w=\sum \frac{\Delta \bar{\varepsilon }}{{\bar{\varepsilon }}_{f}}w$$

where $\Delta \bar{\varepsilon }$ is the equivalent plastic strain increment and ${\bar{\varepsilon }}_{f}$ is the equivalent fracture strain. Damage begins to initiate when *w* equals to 1. Equation (3) shows the general expression for the JC fracture model.

(3) $${\bar{\varepsilon }}_{f}=\left[D_{1}+D_{2}\exp\left(D_{4}\ln\frac{\dot{\bar{\varepsilon }}}{{\dot{\bar{\varepsilon }}}_{0}}\right)\right]\left[1+D_{5}\frac{T-T_{r}}{T_{m}-T_{r}}\right]$$

where *P* is the hydrostatic pressure.

The five JC fracture model parameters *D_i_* (*i* = 1, 2, 3, 4, 5) of Inconel 718 are presented in Table 3.

An energy-based ductile fracture criterion was adopted to describe material damage evolution in a finite element analysis [26]. The parameter of fracture energy *G_f_* expressed in Equation (4) denotes the energy needed to form a unit area crack.

(4) $$G_{f}=\int_{{\bar{\varepsilon }}_{0}}^{{\bar{\varepsilon }}_{f}}L\sigma_{y}d\bar{\varepsilon }=\int_{0}^{{\bar{u}}_{f}}\sigma_{y}d\bar{u}$$

where ${\bar{\varepsilon }}_{0}$ is the initial plastic strain and ${\bar{u}}_{f}$ is the equivalent fracture displacement. Linear damage evolution was considered, and the damage variable *D* was calculated with Equation (5).

(5) $$D=\frac{L\bar{\varepsilon }}{{\bar{u}}_{f}}=\frac{\bar{u}}{{\bar{u}}_{f}}$$

The material stiffness will be fully decreased when the parameter *D* evolves to 1; after that, the involved elements will be deleted and the crack will be generated.

## 3. Results and Discussions

### 3.1. Geometrical Characteristics of Chips

**Figure 2:**
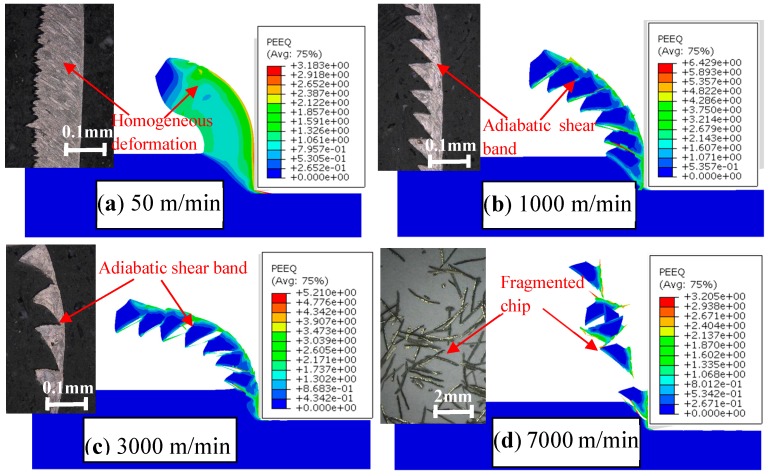
Different chip morphologies produced under varied cutting speeds for Inconel 718.

The chip morphology of Inconel 718 produced within the cutting speed range of 50–7000 m/min is exhibited in Figure 2, in which the contours of the equivalent plastic strain (PEEQ) within the chip deformation areas are shown clearly. Three different chip morphologies, a continuous one, a serrated one, and a fragmented one, occur within the investigated cutting speed range for Inconel 718. It can be figured out from Figure 2a that a continuous chip is formed at *V* = 50 m/min, while the chip morphology transforms to a serrated one while the cutting speed improves until a fragmented chip forms at *V* = 7000 m/min. Meanwhile, the reliability of the simulation results has been validated with orthogonal cutting experiments. The detailed experimental procedures can be tracked in the literature [9]. Figure 2 also presents that homogeneous material deformation occurs within the continuous chip while inhomogeneous deformation occurs within the serrated chip. Periodic adiabatic shear bands with severe localized deformation appear in the serrated chips as indicated in Figure 2b,c.

Figure 2 indicates that the PEEQs within the primary deformation zone and tool–chip interface are much larger than those in other areas due to the localized shear deformation in these two regions. It can also be figured out that the strain to material fracture within the serrated chip has negative correlation with the cutting speed. The largest strain within the PEEQ contours declines from 6.429 to 3.205 when the cutting speed changes from *V* = 1000 m/min to *V* = 7000 m/min as shown in Figure 2b–d. The reason leading to this phenomenon is that embrittlement of the workpiece material occurs under ultra-high cutting speeds, which has been investigated intensively in our previous work [27].

**Figure 3:**
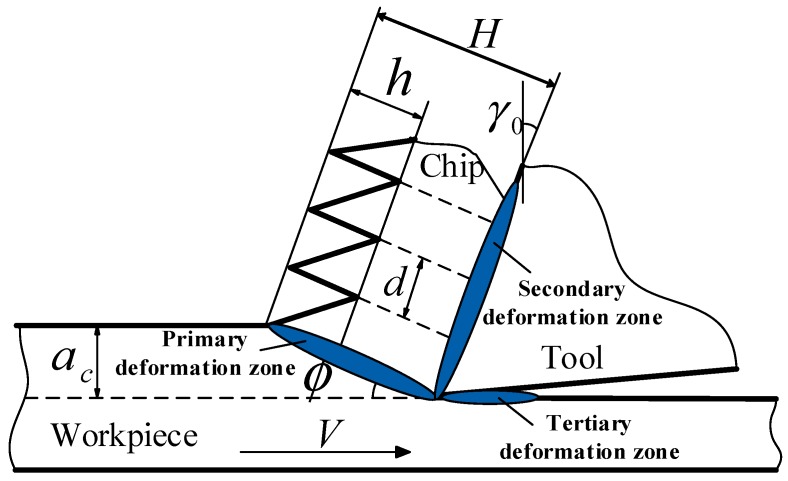
Diagram of serrated chip formation during orthogonal cutting.

The serrated chip formation of Inconel 718 can be described schematically in Figure 3. Three deformation zones are presented by the shadow areas. The geometrical features of serrated chips can be characterized quantitatively with serrated degree *G_s_* and serrated frequency *f*. The two parameters can be calculated with Equations (6) and (7), respectively.

(6) $$G_{s}=\frac{h}{H}$$

where *h* and *H* are the heights of the discontinuous part and the whole part of serrated chip, respectively, as exhibited in Figure 3.

(7) $$f=\frac{Va_{c}}{d\left(H-h/2\right)}$$

where *d* is the distance of two adjacent chip segments as exhibited in Figure 3.

**Figure 4:**
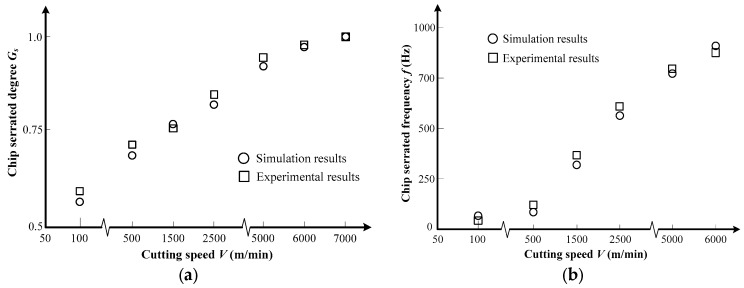
Variation of chip geometrical characteristics versus cutting speed for Inconel 718 (**a**) chip serration degree; (**b**) chip serration frequency.

Figure 4 shows the variation of chip serration degree and serration frequency versus cutting speed. Figure 4a demonstrates that the increase in cutting speed results in an increase of serration degree *G_s_*. The parameter *G_s_* evolves to one under *V* = 7000 m/min, which means that the neighboring chip segments separate completely and fragmented chips form as shown in Figure 2.

The variation of chip serration frequency under different cutting speeds is illustrated in Figure 4b. The results show that the increase in cutting speed leads to an increase of chip serration frequency. Similar trends between the cutting speed and chip morphology, including the chip serration degree and serration frequency, have also been found in the publications of other researchers [28,29]. However, when the chip morphology evolves to a fragmented one at *V* = 7000 m/min, the parameter *f* is no longer applicable to characterize the chip geometrical features. The agreement between the experimental and simulation results of chip geometrical features has also been demonstrated in Figure 4.

### 3.2. Chip Deformation and Fracture under Different Cutting Speeds

**Figure 5:**
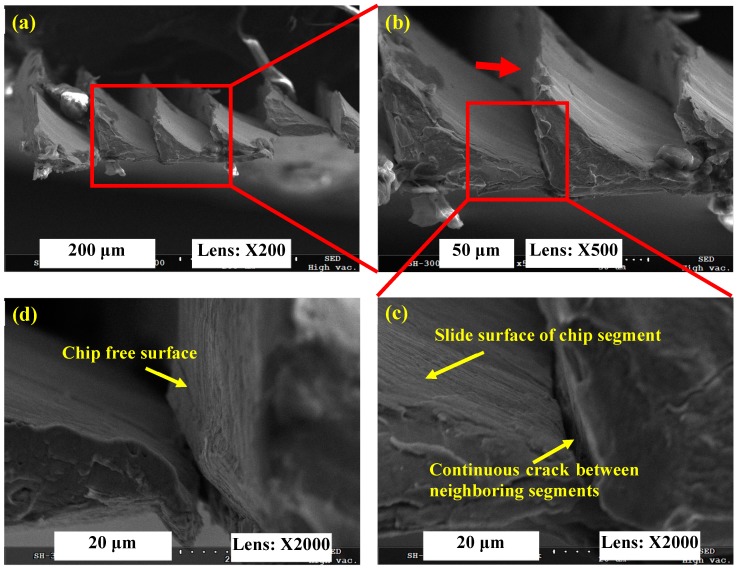
SEM micrographs of a serrated chip for Inconel 718 produced under *V* = 6000 m/min. (**a**) Transverse fracture surface of serrated chip; (**b**,**c**) indicate microscopic morphologies of magnified areas shown in (**a**,**b**); (**d**) indicates a chip’s free surface morphology observed from the arrow direction in (**b**).

Chip fracture surface morphologies were observed to research the chip deformation behavior for Inconel 718 under varied cutting speeds using a scanning electron microscope (SEM). Figure 5 shows the SEM micrographs of a serrated chip for Inconel 718 produced under *V* = 6000 m/min. The results in Figure 5a,b indicate that the conjunctive area of neighboring chip segments is small under *V* = 6000 m/min. The chip serration degree approaches 1, and nearly detached chip segments are produced. Figure 5c presents the micrograph of a chip segment slide surface, which is relatively smooth and has been formed by shear deformation and ductile fracture. Meanwhile, a continuous crack between neighboring segments can be observed. When a continuous crack propagates thoroughly from a chip’s free surface to the bottom surface with a further increase of cutting speed, completely separated chip segments will be formed. A chip’s free surface was also observed as shown in Figure 5d from the view direction as shown by the arrow in Figure 5b.

**Figure 6:**
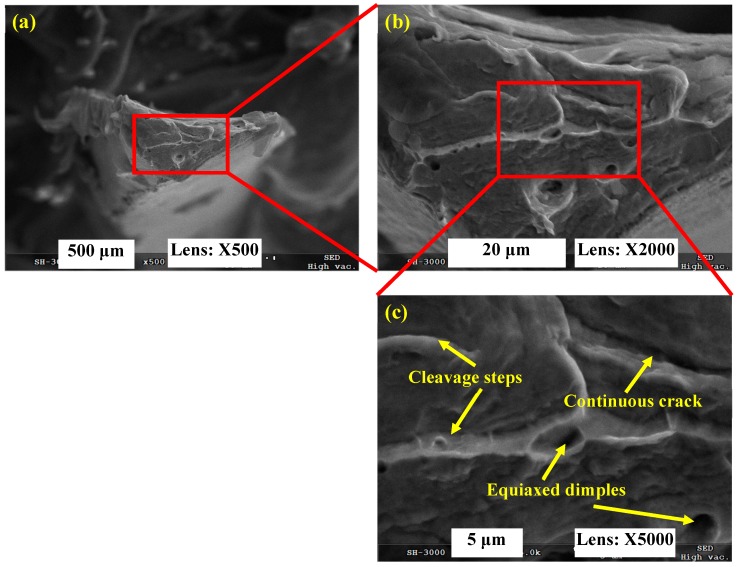
Fracture surface morphology of fragmented chip for Inconel 718 produced under *V* = 7000 m/min. (**a**) Transverse fracture surface of fragmented chip; (**b**,**c**) indicate microscopic morphologies of magnified areas shown in (**a**,**b**).

The transverse fracture surface of the fragmented chip produced under *V* = 7000 m/min was observed as shown in Figure 6. It can be figured out that the micrographs of the fragmented chip are distinctly different compared with the serrated chip as shown in Figure 5. Cleavage steps, equiaxed dimples, and continuous cracks with a river pattern are observed over the chip’s transverse fracture surface. All of these fracture morphologies are typical brittle fracture characteristics, which indicate that fragmented chips are generated through brittle fracture rather than plastic deformation and ductile fracture. Embrittlement of the workpiece material under ultra-high cutting speeds leads to the variation in chip deformation behavior, which then determines chip formation with different morphologies.

**Figure 7:**
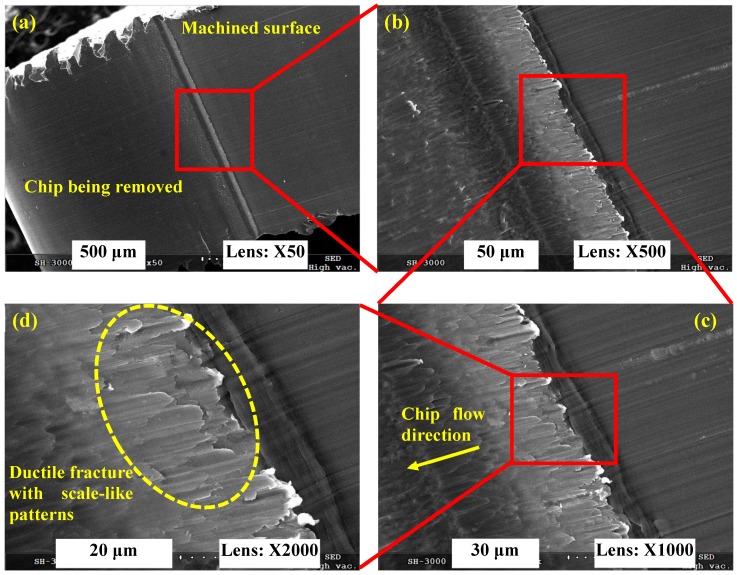
SEM micrographs of chip root for Inconel 718 produced under *V* = 1000 m/min. (**a**) Macroscopic morphology of chip root; (**b**–**d**) indicate microscopic morphologies of magnified areas shown in (**a**–**c**).

**Figure 8:**
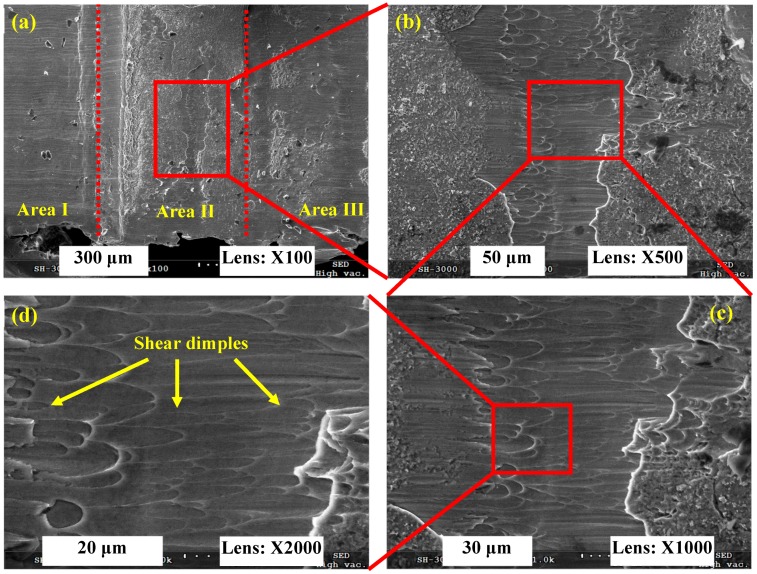
SEM micrographs of a chip root for Inconel 718 produced under *V* = 6000 m/min. (**a**) Macroscopic morphology of chip root; (**b**–**d**) indicate microscopic morphologies of magnified areas shown in (**a**–**c**).

**Figure 9:**
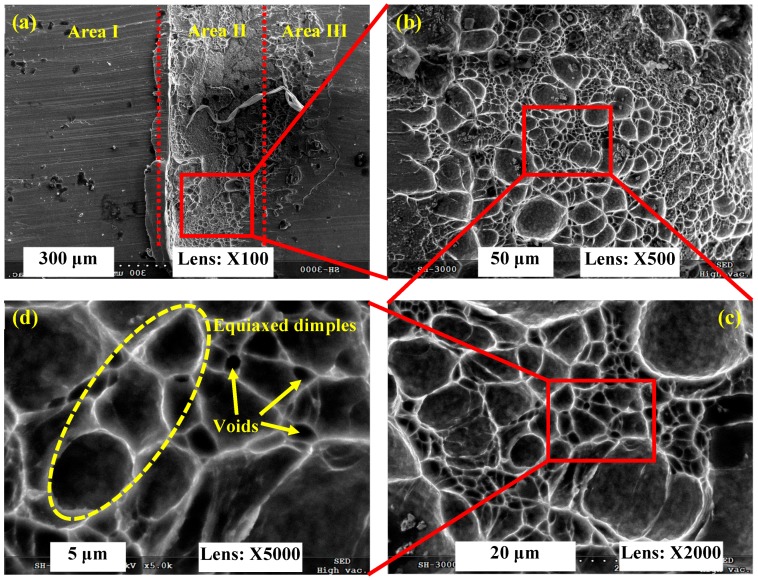
SEM micrographs of a chip root for Inconel 718 produced under *V* = 7000 m/min. (**a**) Macroscopic morphology of chip root; (**b–d**) indicate microscopic morphologies of magnified areas shown in (**a**–**c**).

In order to obtain deeper insights into the chip deformation mechanism, chip roots were gained under varied cutting speeds with the method proposed by Buda [30]. Chip roots freeze material deformation at the moment a chip is removed, which provides more information for understanding the chip formation process. The chip roots produced under *V* = 1000 m/min, 6000 m/min, and 7000 m/min are presented in Figure 7, Figure 8 and Figure 9, respectively. Figure 7a shows that the chip being removed is attached to the machined surface under *V* = 1000 m/min. The chip flow direction can be distinguished from the elongated material direction as shown in Figure 7c. With high-magnification SEM micrographs, a ductile fracture surface with scale-like patterns can be seen clearly within the separation area between the chip being removed and the machined surface as shown in Figure 7d. It can be deduced that plastic deformation combined with ductile fracture is the formation mechanism for a serrated chip.

Figure 8 presents the SEM micrographs of a chip root for Inconel 718 produced under *V* = 6000 m/min. Because the conjunctive area of neighboring chip segments is small (as shown in Figure 5), which leads to low connective strength between the chip being removed and the machined surface, there is no chip being formed that is adhered to the machined surface as displayed in Figure 8a. There are three separated areas: the machined surface area (Area I), the material being removed area (Area II), and the un-machined surface area (Area III) as shown in Figure 8a. The material being removed area represents the chip root. Large amounts of shear dimples are distributed in the chip being removed area, which indicates that shear deformation occurs during chip formation for Inconel 718 under *V* = 6000 m/min. The elongation direction of the shear dimples is in line with the shear force imposed by the cutting tool.

The chip root morphology of Inconel 718 produced under *V* = 7000 m/min as shown in Figure 9 is distinctly different compared to those produced under *V* = 1000 m/min and 6000 m/min as shown in Figure 7 and Figure 8, respectively. Because the fragmented chips are generated under *V* = 7000 m/min, there is also no attached chip on the finished surface as shown in Figure 9a. It can be seen that there are also three separated areas, including the machined surface area (Area I), the material being removed area (Area II), and the un-machined surface area (Area III). For the chip root area displayed in Area II, a mass of equiaxed dimples are observed on the fracture surface. From the high-magnification SEM micrograph shown in Figure 9d, lots of voids can be seen among the equiaxed dimples. When adjacent dimples or voids grow and assemble together, a continuous crack will be formed and lead to chip fragment formation. It demonstrates that the formation mechanism of a fragmented chip is brittle fracture rather than plastic deformation and ductile fracture.

### 3.3. Influence of JC Constitutive Parameters on Chip Shear Localization

The mechanical properties of the workpiece material are the intrinsic factors that affect material deformation and fracture. Equations (1) and (3) indicate that the material constitutive and fracture parameters play a significant role in material removal and chip formation. The parameter of shear localization sensitivity is proposed to assess the effects of material mechanical properties on serrated chip formation quantitatively. Through changing the material constitutive and fracture parameters within a given range separately, variations of chip serration degree are obtained and compared quantitatively. The chip serration degree produced with the original material parameters is regarded as the reference value. The cutting speed *V* is kept constant as 1000 m/min in Section 3.3 and Section 3.4.

When the material constitutive or fracture parameters are varied, the differences between the calculated serration degrees and the reference value are obtained. Then, the difference can be characterized with the parameter *S*, which is called shear localization sensitivity, and is expressed by Equation (8).

(8) $$S=\frac{G_{S}^{D_{i}}-G_{S}^{original}}{G_{S}^{original}}\times 100\%$$

where $G_{S}^{D_{i}}$ is the chip serration degree calculated with the investigated constitutive or fracture parameters and $G_{S}^{original}$ is the referenced value.

To research the effects of JC constitutive parameters on the chip formation of Inconel 718 in a large scope, the researched parameters are varied within a scope of a twenty percent decline to a twenty percent improvement on the basis of the original values. The varied interval of the researched constitutive parameters is set as ten percent of the original ones. When a researched constitutive parameter is varied, the other ones remain unchanged. The details of the researched scopes of the JC constitutive parameters for Inconel 718 are shown in Table 4.

**Figure 10:**
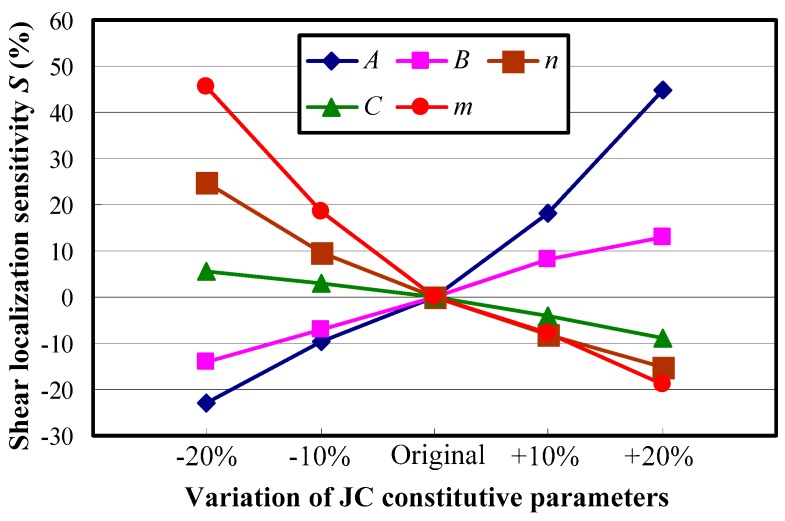
Influences of material constitutive parameters on chip shear localization for Inconel 718.

Figure 10 presents the shear localization sensitivity *S* of serrated chips under varied constitutive parameters for Inconel 718. The results show that with the initial yield stress *A* or the hardening modulus *B* changing from a twenty percent decline to a twenty percent improvement, the shear localization sensitivity transforms from negative to positive. It indicates that the chip serration degree enhances with an increase in the constitutive parameter *A* or *B*. This phenomenon can be attributed to the varied relationship between the mechanical and thermal loadings caused by different mechanical properties of the workpiece material. When the parameter *A* or *B* increases, the material strength or hardening modulus improves. It will then cause much more cutting energy consumption when the same plastic deformation occurs for the workpiece material, and more heat is produced in the chip deformation area through plastic work transformation. As a result, the localized shear deformation and consequent ductile fracture are enhanced due to the more prevailing role of the thermal softening rather than hardening effects. Comparatively, the other three constitutive parameters of *n*, *C*, and *m* all have negative relationships with the shear localization sensitivity. The reason is that the material hardening capacity improves with an increase in these parameters, which restrains the development of localized shear deformation to some extent.

The processing methods that can improve the material yield stress or the hardening modulus mainly include heat treatment processing (e.g., quenching, surface carburizing, and surface nitriding), plastic deformation processing, and the addition of alloying elements [23]. Based on different reinforcement principles, these processing methods can be divided into solution strengthening, dispersion strengthening, and deformation strengthening. The principles of heat treatment processing and the addition of alloying elements mainly belong to solution strengthening and dispersion strengthening. Deformation strengthening is applied to increase the dislocation density within a workpiece material, which promotes resistance to dislocation movement and increases plastic deformation resistance. Based on the adiabatic shear theory for serrated chip formation, the factors that are beneficial for thermal softening can increase chip serration.

It can also be seen from Figure 10 that the effects of material constitutive parameters *A* and *m* on chip shear localization are much more remarkable than the other parameters. When the initial yield stress *A* varies within the whole researched scope, the chip shear localization sensitivity increases from −23% to 44.7%. When the thermal softening coefficient *m* changes in the same range, the chip shear localization sensitivity changes from 45.7% to −18.9%. The effect of the strain rate dependency coefficient *C* on chip shear localization is the weakest. The shear localization sensitivity only varies less than 15% when the parameter *C* changes within the whole researched scope. Agmell et al. [31] and Yaich et al. [32] also found that the material constitutive parameters of the initial yield stress *A* and the thermal softening coefficient *m* have a more predominant influence than the other parameters on the chip thickness ratio and maximum strain in cutting zones, which demonstrates the great significance of the two constitutive parameters of *A* and *m*.

### 3.4. Influence of JC Fracture Parameters on Chip Shear Localization

To research the effects of material fracture parameters on chip formation within a large scope, the JC fracture parameters were varied in the range of a one hundred percent decline to a one hundred percent improvement on the basis of the original values. The varied interval of the researched fracture parameters is set as half of the original ones. Because the base values of material fracture parameters are much smaller than those of the constitutive parameters, the variations of the researched JC fracture parameters are larger than those of the constitutive parameters. The details of the researched JC fracture parameters for Inconel 718 are presented in Table 5.

**Figure 11:**
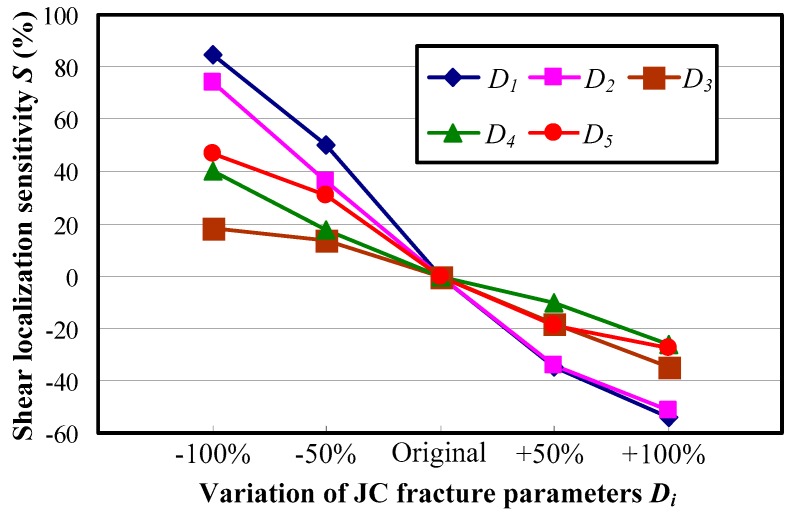
Influences of material fracture parameters on chip shear localization for Inconel 718.

Figure 11 shows the shear localization sensitivity *S* under varied material fracture parameters for Inconel 718. The results show that with a decline in all five material fracture parameters, the shear localization sensitivity *S* is positive while it changes to negative when the researched material fracture parameters increase. The positive value of *S* indicates an enhancement of chip shear localization and an increase in chip serration, while the negative value of *S* indicates the opposite change of chip morphology. The reason is that the material fracture strain ${\bar{\varepsilon }}_{f}$ increases with all researched fracture parameters as expressed by Equation (3). The resistance to fracture will be promoted when the fracture strain of the workpiece material increases, which then leads to the suppression of chip serration.

It can be figured out from Figure 11 that the JC fracture parameters *D*_1_ and *D*_2_ play much more remarkable roles in chip shear localization than the other ones regardless of whether the effect is promotion or suppression. Comparatively, the influence of material fracture parameter *D*_3_ on chip localized shear deformation is the weakest, and the effects of parameters *D*_4_ and *D*_5_ fall in between.

**Figure 12:**
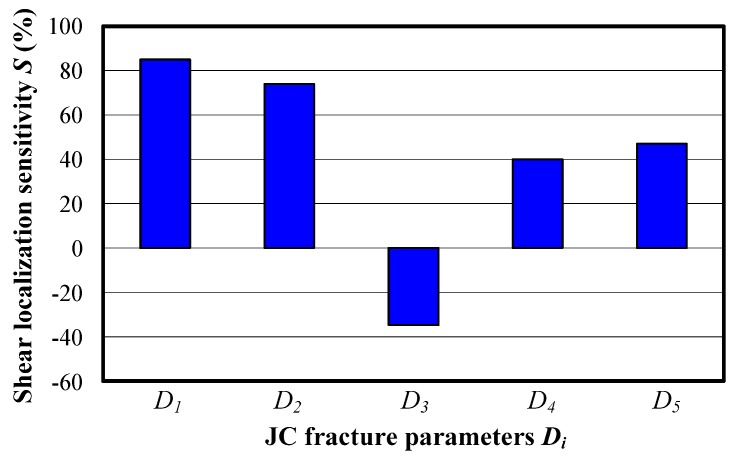
Variations of chip shear localization when the respective fracture parameter *D_i_* equals to zero.

The individual effects of strain, strain rate, and temperature on the chip localized shear deformation are analyzed through setting a certain JC fracture parameter to be zero. With this method, the separated influences of initial fracture strain, stress state, strain rate, and temperature on the chip localized shear deformation for Inconel 718 are achieved as shown in Figure 12. The results show that with the condition of *D*_3_ = 0, the chip localized shear deformation is repressed while it is facilitated when the other four fracture parameters are varied to zero. In addition, the parameter *S* is much larger when the parameter *D*_1_ or *D*_2_ is zero, which illustrates the great influence the initial fracture strain and the stress state have on serrated chip formation. Yaich et al. [32] also drew the conclusion that the parameters *D*_1_ and *D*_2_ have a more remarkable influence on the chip formation process than the other fracture parameters considering the variation of cutting force under different material fracture parameters. The results illustrate that research on stress states within cutting zones has vital importance for the analysis of the chip formation process.

## 4. Conclusions

The paper has researched the influences of material mechanical properties on serrated chip formation for Inconel 718. The parameter of shear localization sensitivity is proposed to characterize the variation of chip localized shear deformation under different material properties. Meanwhile, finite element analyses for high-speed orthogonal cutting of Inconel 718 were performed and validated with experimental results. The deformation behavior and the fracture behavior of chips and chip roots for Inconel 718 have also been researched. The research provides guidance for controlling chip formation through the adjustment of material mechanical properties and the selection of appropriate cutting parameters. The main conclusions can be drawn as follows:

1. The material constitutive parameters of initial yield stress *A* and hardening modulus *B* have positive relationships with the chip localized shear deformation, while the parameters *n*, *C*, and *m* all have the opposite influence on chip shear localization. The effects of parameters *A* and *m* on chip localized shear deformation are much more remarkable than the other three parameters. With the parameter *A* or *m* varying from a 20% decrease to a 20% increase, the chip shear localization sensitivity changes by 67.7% and 64.6%, respectively.
2. When the JC fracture parameters of the workpiece material decline, the chip localized shear deformation is promoted while it is repressed when the material fracture parameters improve. The results demonstrate that the influences of parameters *D*_1_ and *D*_2_ on chip localized shear formation are more remarkable than the other three parameters. The chip shear localization sensitivity is larger than 70% when the parameter *D*_1_ or *D*_2_ is zero.
3. The research demonstrates that an increase in the cutting speed promotes chip serration in Inconel 718 until the chip morphology becomes fragmented at the critical cutting speed of 7000 m/min. Serrated chips are generated by the mechanism of plastic deformation combined with ductile fracture, while fragmented chips are generated by brittle fracture due to embrittlement of the workpiece material under ultra-high cutting speeds.
4. The limitation of this paper is that the influences of material mechanical properties on the chip formation process are researched only with JC material models and only the orthogonal cutting method has been focused on. The influence of other material models on the chip formation process and chip formation in more complex cutting modes will be researched in the future.

## Figures and Tables

**Table 1:** Johnson–Cook (JC) constitutive parameters of Inconel 718 [22].

*A* (MPa)	*B* (MPa)	*n*	*C*	*m*
1290	895	0.526	0.016	1.55

**Table 2:** Physical and mechanical properties of Inconel 718 [23].

Density (kg/m^3^)	Elastic Modulus (GPa)	Poisson’s Ratio	Thermal Conductivity (W/m·K)	Specific Heat (J/kg·K)	Thermal Expansion Coefficient × 10^−6^ (/K)	Melting Temperature (K)
8240	200 (93 K) 181 (573 K) 160 (773 K) 141 (973 K)	0.3 (293 K) 0.3 (473 K) 0.31 (691 K) 0.32 (873 K)	10.63 (293 K) 14.7 (373 K) 17.8 (573 K) 19.6 (773 K)	435 (293 K) 481.4 (573 K) 514.8 (773 K) 573.4 (973 K)	11.8 (293 K–373 K) 13.0 (293 K–573 K) 14.1 (293 K–673 K) 14.8 (573 K–873 K)	1573

**Table 3:** JC fracture model parameters of Inconel 718 [25].

*D*_1_	*D*_2_	*D*_3_	*D*_4_	*D*_5_
0.04	0.75	−1.45	0.04	0.89

**Table 4:** The researched scope of constitutive parameters for Inconel 718.

Parameters	↓ 20%	↓ 10%	Original	↑ 10%	↑ 20%
*A*	1032	1161	1290	1419	1548
*B*	716	805.5	895	984.5	1074
*n*	0.4208	0.4734	0.526	0.5786	0.6312
*C*	0.0128	0.0144	0.016	0.0176	0.0192
*m*	1.24	1.395	1.55	1.705	1.86

**Table 5:** The researched JC fracture parameters for Inconel 718.

*D_i_*	↓ 100%	↓ 50%	Original	↑ 50%	↑ 100%
*D*_1_	0	0.02	0.04	0.06	0.08
*D*_2_	0	0.375	0.75	1.125	1.5
*D*_3_	−2.9	−2.175	−1.45	−0.725	0
*D*_4_	0	0.02	0.04	0.06	0.08
*D*_5_	0	0.445	0.89	1.335	1.78
